# Influence of Current on Soil Corrosion of Galvanized Steel of Grounding Grids

**DOI:** 10.3390/mi13020190

**Published:** 2022-01-26

**Authors:** Linbo Song, Cheng Zhang, Jing Zhao, Rui Yang, Yuan Yuan

**Affiliations:** 1 College of Materials Science and Engineering, Chongqing University, Chongqing 400044, China; 202109021149t@cqu.edu.cn (L.S.); 20163014@cqu.edu.cn (C.Z.); 2 State Grid Chongqing Electric Power Research Institute, Chongqing 401123, China; zhaojing_sgcc@163.com (J.Z.); yangrui1@cq.sgcc.com.cn (R.Y.)

**Keywords:** galvanized steel, grounding grids, corrosion behavior, electric current

## Abstract

Grounding grid materials are vulnerable to soil corrosion, which is detrimental to the safe operation of the grounding grids and even lead to serious accidents of power transmission. In this paper, galvanized steel was used as the typical grounding grid material which was buried in the soil and then electrified with AC and DC current for two weeks. The corroded samples under different current conditions were characterized and compared. The experimental results show that the corrosion degree of galvanized steel gradually aggravated with the increasing of the current, especially under DC current. Further, the mechanism of the influence of current on soil corrosion is explored. It is found that under the same magnitude of current, the corrosion degree of galvanized steel under DC current is greater than that under AC current.

## 1. Introduction

Grounding grids are important to ensure the safety of current transmission. When short circuits are caused, they have the function of maintaining voltage balance and releasing electric current [1,2]. Galvanized steel has been widely used as a kind of material for ground grids in power grids and for power plant equipment [3,4,5]. However, galvanized steel will be buried in the soil and electrified due to the working conditions of power systems, which will speed up the corrosion of the galvanized steel, causing it to be damaged [6,7]. When the galvanized steel of the grounding grid fails due to corrosion, the current cannot be spread to the earth in time. In this case, the equivalent resistance and potential of the soil increase greatly, and the voltage in the secondary circuit also increases correspondingly, resulting in the failure of the transmission line, even endangering human safety and causing major safety accidents and huge economic loss [2,3,8].

The research on the corrosion of grounding grid materials has a history of more than half a century. As early as the 1950s, scientists began to research corrosion detection and protection of grounding grids [8,9,10,11,12,13]. Rajan et al. [14] studied the relationship between corrosion and grounding. On this basis, he presented the necessary principles and security considerations for the design of grounding grid. Lawson et al. [15] gave four test methods to test the corrosion rate of grounding grid materials. Some important formulas are given. However, until now, the corrosion problem of grounding grid materials has not been solved perfectly. In recent years, the loss caused by the corrosion of carbon steel is increasing, and more projects choose galvanized steel as grounding grid material. Compared with carbon steel, galvanized steel has the advantages of simpler manufacturing process, lower cost and better corrosion resistance. The zinc layer on the surface of the galvanized steel plate can be preferentially corroded to form a dense oxide film to protect the internal steel substrate from corrosion.

At present, galvanized steel has become one of the main materials of grounding grids, and scientists have paid considerable attention to the corrosion of galvanized steel. Morales et al. [16,17] made statistics on the corrosion of galvanized steel in the tropical environment, and gave the change of the corrosion rate of galvanized steel with temperature. Portella et al. [18] conducted a lot of research on the corrosion of galvanized steel in the atmosphere, and found that acid substances (Cl-, NOx, CO2, etc.) are the main factors affecting the corrosion of galvanized steel, especially in the industrial environment of transmission and transformation stations, power plants and so on [19,20,21]. So, the corrosion behavior of galvanized steel is affected by many factors.

With the continuous transformation and development of power transmission, ultra-high voltage and ultra-long distance transmission is becoming more and more popular [22,23]. Therefore, safety operation has become the hot topic. To ensure the safe operation of power transmission, higher requirements are put forward for the materials of ground grids. Soil corrosion is the key and difficulty to threaten the safe operation of grounding grid [5]. In operating conditions, grounding grid materials need to be applied with electric current in corrosive soil environment, which increases the rate of corrosion and leads to material failure. In previous studies, there were few studies on the corrosion behavior and corrosion mechanism of galvanized steel of grounding grids materials under working conditions. In this paper, galvanized steel was used as the experimental material. The galvanized steel was buried in the actual soil and electrified with different types and different sizes of electric current. In the actual working environment, the corrosion process of grounding grid materials is not easy to find. So, we have creatively built a simplified working environment for grounding grid materials to observe the corrosion process. Unlike other scholars, we researched the variation rules of DC and AC currents and compared them, which made our results completer and more comprehensive. Finally, the law of the change of the corrosion behavior of galvanized steel with electric current is found and the corrosion mechanism is explored. It is expected to provide help for further solving the problems of corrosion and material selection of grounding network equipment.

## 2. Materials and Methods

The galvanized steel used in this experiment is provided by Chongqing Iron and Steel Co., LTD (Chongqing, China) and cut into 50 × 30 × 4 mm. The thickness of zinc layer of galvanized steel is 770 μm. Soil (shown as Table 1) from Fuling Station in Chongqing was selected. The experiment also required alcohol provided by Chongqing Wan sheng East Sichuan Chemical Co. LTD (Chongqing, China) and distilled water.

**Figure 1:**
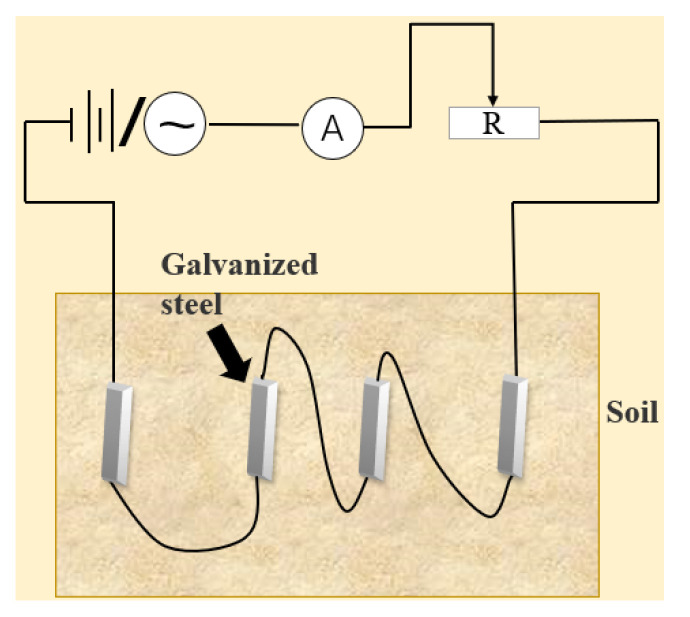
Scheme diagram of the experimental work.

The following chart explains the experimental procedure carried out through this work (Figure 1).

Before the experiment, the samples were divided into seven groups and buried in the experimental soil. In order to ensure that corrosion is only affected by current size and type, we control the experiment with only current as a single variable, and each group of current is 0 mA/0.5 mA/50 mA/300 mA (AC/DC). After two weeks, seven groups of samples were taken. Remove corrosion products according to GB/T16545-1996 standard [24]. 17.6 g ammonium chloride and 100 mL distilled water were mixed to prepare the mixed solution, and the sample was immersed in the mixed solution at 80 °C for 30 min to achieve the purpose of rust removal. After rust removal, wash with distilled water and then rinse with alcohol. They are dried in a drying oven and weighed.

The surface morphology and elemental analysis were characterized using the field emission scanning electron microscope system (SEM), the energy dispersive spectrometer (EDS) provided by Carl Zeiss Management Co. LTD (Shanghai, China) and the X-ray diffractometer (XRD) provided by Chengdu Yuke Technology Development Co., LTD (Chengdou, China).The electrochemical impedance test was carried out by electrochemical workstation provided by Shanghai chenhua Instrument Co. LTD (Shanghai, China), using a three-electrode system, the working electrode was corroded by different currents of galvanized steel, the reference electrode was saturated calomel electrode (SCE), the auxiliary electrode was Pt electrode, and 0.1 mol/L NaCl solution was used as the electrolytic solution. The frequency range of electrochemical impedance spectroscopy (EIS) was 0.01–10^5^ Hz, and the AC excitation signal was 10 mV.

## 3. Result and Discussion

### 3.1. Morphology and Structure

The corrosion degree can be judged by watching the surface macromorphology of galvanized steel. The magnification of the photographs is 1. The corroded experimental samples and original sample was presented in Table 2. compared the surface macromorphology of galvanized steel, it is found that the current will aggravate the corrosion of galvanized steel. Under the AC current, the corroded area gradually increases with the increase of current, and the corroded area is concentrated at both ends of galvanized steel. Under the DC current, the corroded area gradually increases with the increase of current, and this trend is much larger than that of AC. The corroded areas are relatively evenly distributed.

**Figure 2:**
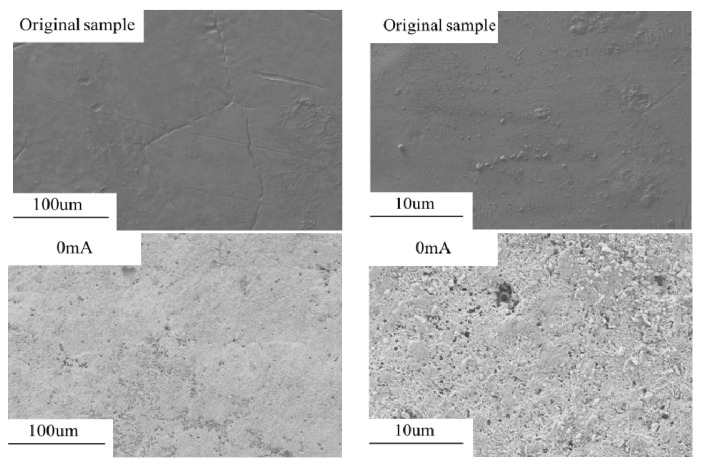

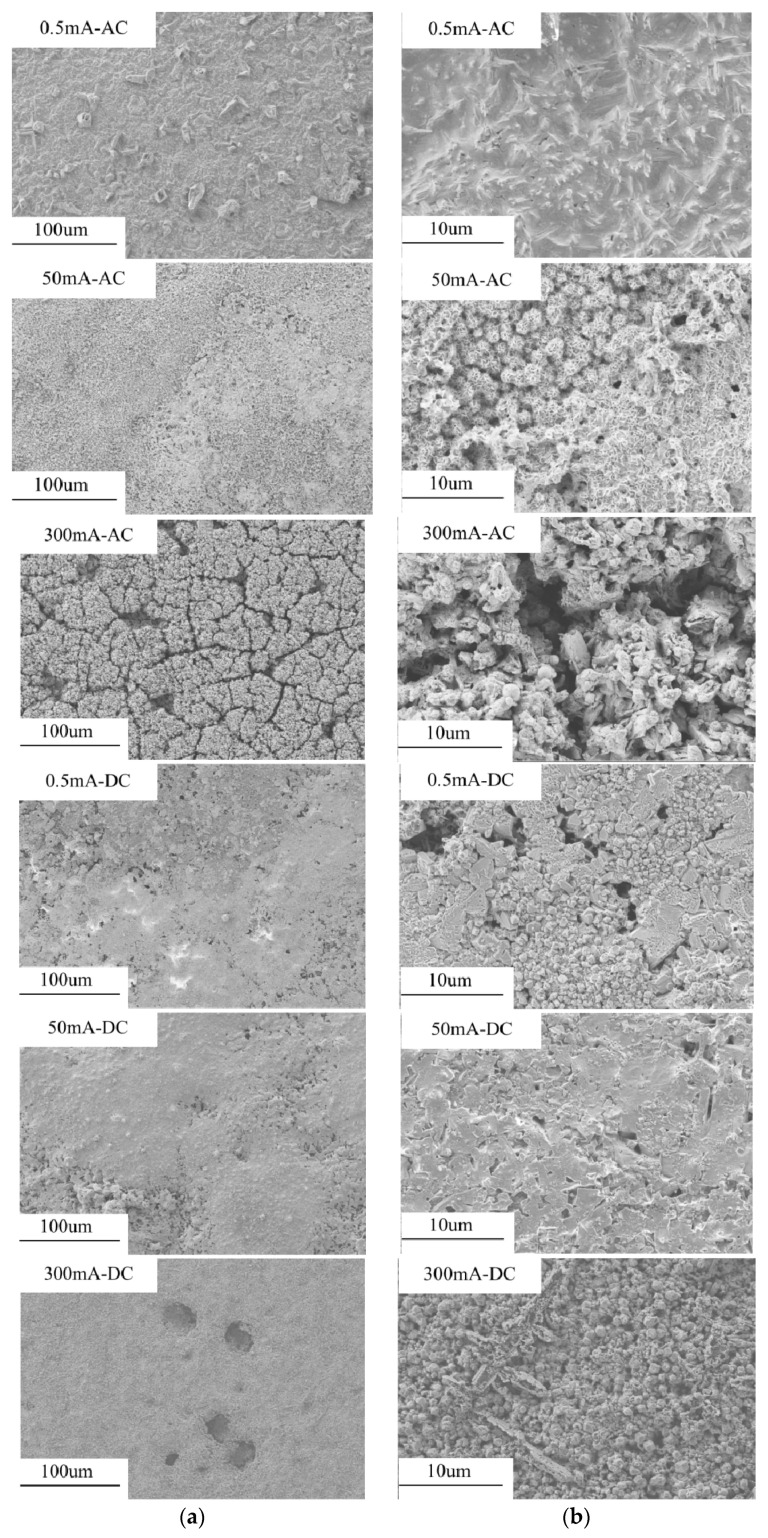
The microscopic morphology of galvanized steel. (**a**) the magnifications is 100, (**b**) the magnifications is 1000.

The results of the SEM are shown in Figure 2. At low magnification (the magnifications is 100), the aperture of small pits on the surface of galvanized steel will gradually increase with the increase of current. Under the DC current, individual and large craters form on the sample surface. Under the AC current, dense pores form on the surface of the sample. As the current increases, the holes gradually form a series of cracks as shown in Figure 2a.

At high magnification (the magnifications is 1000), it can be seen that under the AC current, the galvanized steel will preferentially form an oxide film to protect the zinc layer from damage (Figure 2b), under the DC current, no oxide film formation is observed. Under the AC current, the oxide film of galvanized steel sample is gradually destroyed with the increase of current, forming porous and loose surface, and finally forming surface cracks. Under the DC current, there are no obvious corrosion products on the surface, and the corrosion surface is smoother than that under the AC current.

**Figure 3:**
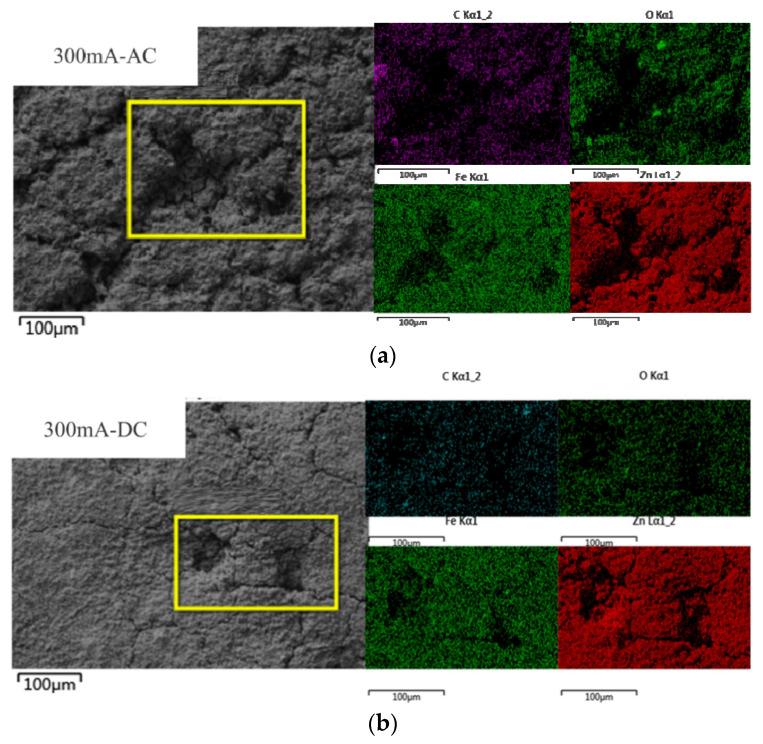
The results of element analysis of galvanized steel. (**a**) 300mA-AC, (**b**) 300mA-DC.

The EDS results are shown in Figure 3. In order to compare the corrosion degree of the DC and AC current, the sample of 300 mA current was selected for measurement. Through the elemental analysis of the corrosion products around the aperture of galvanized steel, it is found that Zn, O, Fe and C elements appeared. Zn is the original element of galvanized steel, the zinc layer is oxidized to ZnO and brought into the O element. The C, Fe elements are unique elements in the inner steel layer, it indicates that the zinc layer was corroded to perforation and the internal steel layer was exposed.

**Figure 4:**
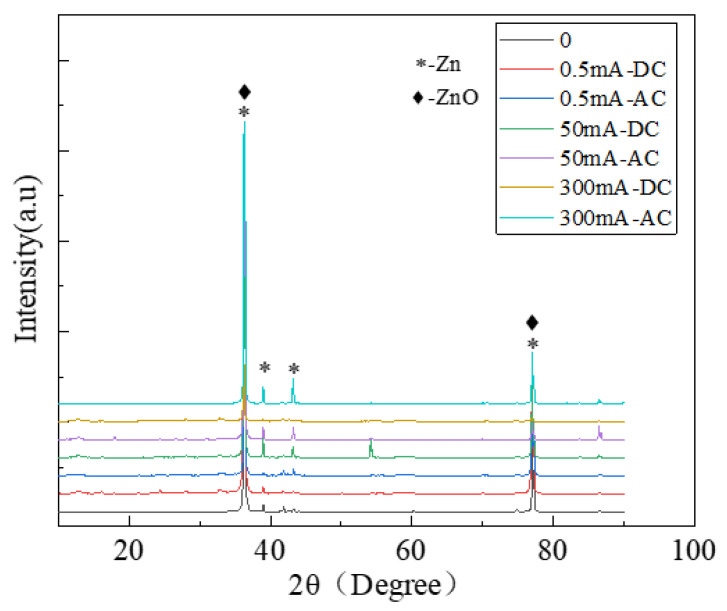
The XRD spectra of galvanized steel.

XRD spectrum results are shown in Figure 4. The diffraction peaks at 2θ angle 36.29°, 38.63°, 43.22° and 77.06° are characteristic peaks of Zn. The diffraction peaks at 2θ angle 36.29° and 77.06° are characteristic of ZnO. It indicates that the corrosion product of the sample after corrosion is ZnO.

### 3.2. Corrosion Behavior and Mechanism

Weightlessness is the most intuitional corrosion behavior data to observe corrosion behavior. The corrosion weight loss rate can be obtained according to Formula (1) to describe the corrosion behavior of galvanized steel. Remove the rust from the samples after two weeks of the experiment. Weigh the weight and record the data. The results are shown in Table 3.

V = W/(S × T) × 106(1)

**Figure 5:**
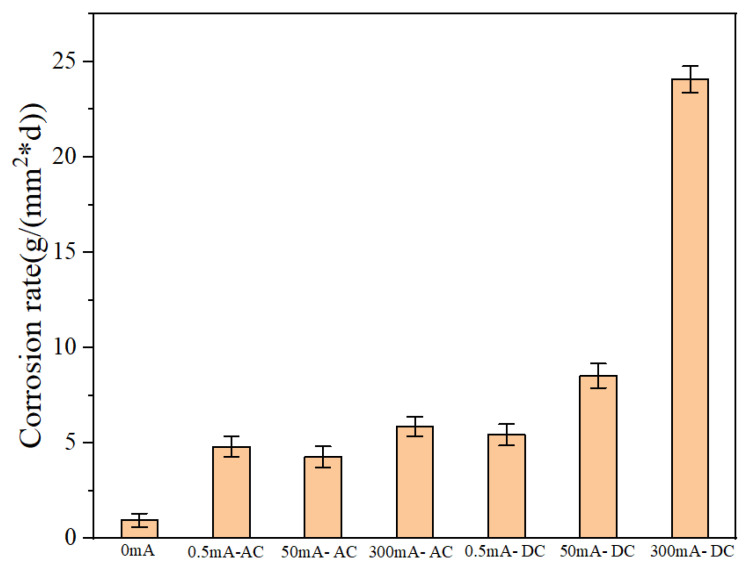
The corrosion rate diagram of galvanized steel.

The weight loss rate can directly and effectively reflect the corrosion degree of the sample. Table 3 shows the weight loss rate of the seven groups of specimens buried in the experimental soil after two weeks. The weight loss rate was plotted as the weight loss rate graph, as shown in Figure 5. From this figure, we can intuitively find that current can significantly increase the corrosion rate of galvanized steel. This phenomenon exists in both DC and AC current. The weight loss rate of galvanized steel increases obviously with the increase of DC current.

**Figure 6:**
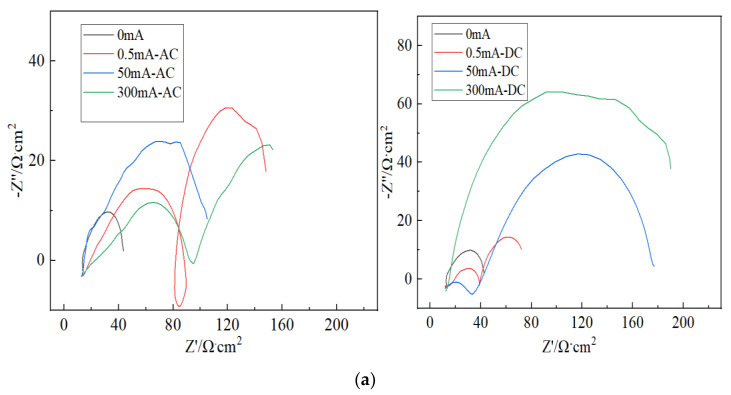

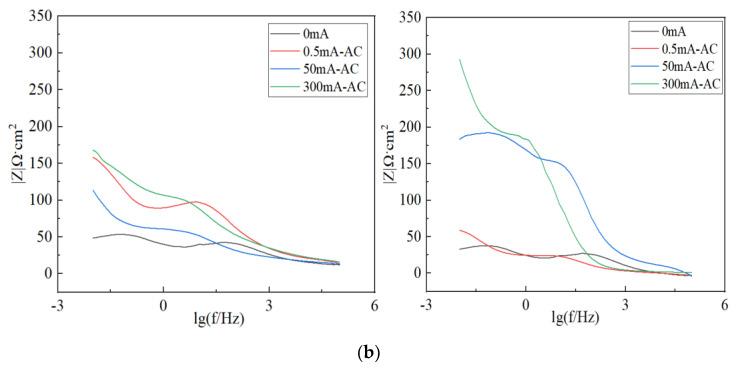
The electrochemical impedance graph of galvanized steel. (**a**) Nyquist figure, (**b**) bode figure.

In order to further investigate the corrosion behavior of galvanized steel, electrochemical impedance test was carried out on the corroded samples. The results are shown in Figure 6. Under the AC current, there are two capacitive loop in the Nyquist figure in 0.5 and 300 mA currents; the radius of capacitive loop is much larger than that of the sample in 0 mA currents, indicating that electrified with AC current can accelerate the formation of oxide film and hinder electron transfer. Under the DC current, there are also two capacitive loops in Nyquist figure in 0.5 and 50 mA currents; with the increase of DC current, the radius of capacitive loop becomes larger and larger, indicating that electrified with DC current can significantly accelerate the degree of galvanized steel corrosion. The capacitive arc diameter of DC power supply is much larger than that of AC power supply under the same current size.

### 3.3. Corrosion Mechanism

Galvanized steel in the soil suffers mainly electrochemical corrosion under the influence of electric current. Galvanized steel with different potentials forms a loop through the corrosive soil medium. The negative polar part of galvanized steel acts as the anode to dissolve zinc layer. The positive polar part is used as the cathode for oxygen reduction reaction [5,25].

**Figure 7:**
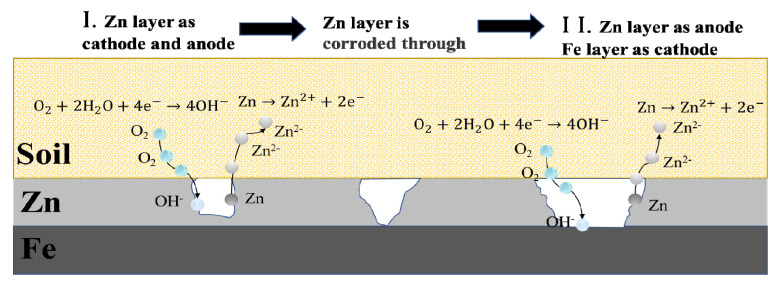
Corrosion mechanism diagram of galvanized steel.

The corrosion mechanism of galvanized steel is shown in Figure 7. The soil corrosion process of galvanized steel can be divided into two stages. In the first stage, the zinc layer acts as both anode and cathode. In this stage, zinc loses electrons and dissolves, and oxygen gains electrons and reduces both occur on the zinc layer. The first stage continues until the galvanizing layer is corroded and perforated to expose the underlying steel substrate. When the underlying steel substrate is exposed to the corrosive medium, the corrosion process enters the second stage. In the second stage, a corrosion cell is formed between the zinc layer and the steel substrate. Due to the negative electrode potential of zinc, zinc as the anode of the corrosion cell is corroded and dissolved, while steel as the cathode occurs oxygen reduction. At this stage the steel is protected as a cathode until all the zinc coating has corroded away.

1. The electrochemical corrosion reaction:

In the first step, as the positive and negative poles of the AC power supply are constantly changing, Zn in the same place can be used as cathode or anode.

Zn as anode:

Zn → Zn^2+^ + 2e^−^ (2)

Zn as cathode:

O_2_ + 2H_2_0 + 4e^−^ → 4OH^−^ (3)

In the second step, Fe and Zn form an electrolytic cell.

Zn as anode:

Zn → Zn^2+^ + 2e^−^ (4)

Most Zn^2+^ will continue to react with OH^−^ in the soil to form hydroxides:

Zn^2+^ + 2OH^−^ → Zn(OH)_2_ → ZnO + H_2_0 (5)

Fe as cathode:

O_2_ + 2H_2_0 + 4e^−^ → 4OH^−^ (6)

**Figure 8:**
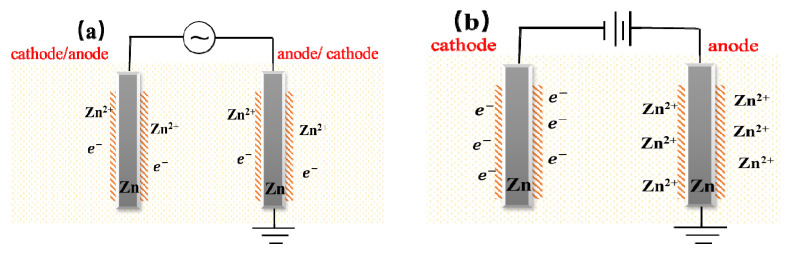
Electrochemical corrosion process: (**a**) under AC current; (**b**) under DC current.

As the corrosion of galvanized steel is an electrochemical reaction process, the part connected to the positive extreme of the power supply has a higher potential, while the part connected to the negative extreme of the power supply has a lower potential, so there is a potential difference between the two. The electrochemical corrosion process of galvanized steel under ac and DC current is shown in Figure 8. 

Under the same type of current, the corrosion degree of galvanized steel increases with the increase of current, especially in the case of DC. The reason is that under the action of current, the outer electrons of Zn ion will be stimulated and easily lose electrons, while the increase of current intensity will increase the potential difference between electrodes, and the outermost electrons will be excited and run out more easily.

Under the same magnitude of current, the corrosion degree of galvanized steel connected to a DC power supply is much greater than that of an AC power supply. The reason for this is that, the escape of outer electrons is a process of energy accumulation, and a certain duration is needed to accumulate energy to excite the outer electrons and to produce Zn^2+^. Under an AC current, AC power is converted from positive to negative electrodes at a fixed frequency, and the anode reaction time is relatively short at this time. Especially when the AC current intensity is low, the outer electrons are less likely to escape. Due to the short time of current stimulating the anode reaction, the effect of AC current on the corrosion process is not as obvious as that of DC current. Under DC current, galvanized steel is not under the action of the power supply with changing polarity. The energy of the reaction will continue to accumulate under the stimulation of the current, so that a large number of zinc atoms at the anode lose electrons to form Zn^2+^, and electrons at the cathode continue to get reduction reaction, which resulting in the corrosion degree of DC current is much higher than that of AC current. 

## 4. Conclusions

The corrosion of grounding grids is one of the factors threatening the normal operation of power transmission. In this paper, galvanized steel was selected as the grounding grid material as the experimental research object, and different current sizes and types of current were passed into the soil to explore the corrosion of different samples. 

From EIS we can find: Under the AC current, the radius of capacitive loop is much larger than that of the sample in 0 mA currents, indicating that electrified with AC current can accelerate the formation of oxide film and hinder electron transfer. Under the DC current, the corrosion products on the electrode surface increase, and a relatively dense oxide film is formed on the electrode surface, which slows down the diffusion of the corrosive medium. The larger DC current, the more obvious the phenomenon; accordingly, the radius of capacitive loop becomes larger and larger. It indicates that electrification with DC current can significantly accelerate the degree of galvanized steel corrosion. The radius of the capacitive loop of a DC power supply is much larger than that of an AC power supply under the same current size. In the Bode diagram, the impedance amplitude increases gradually as the current increases, which can also confirm the above conclusion. Considering the corrosion rate, macroscopic diagrams, and SEM, it was found simultaneously that: under the same current type, the corrosion degree of galvanized steel increases with the increase of current, especially in the case of a DC power supply; under the same amount current, the corrosion degree of galvanized steel connected to a DC power supply is greater than that of an AC power supply, and we explained the mechanism. In addition, the XRD patterns show that the corrosion products of galvanized steel are ZnO.

## Figures and Tables

**Table 1:** Experimental soil parameter.

Parameter Types	Numerical
Cl^−^ (mg/kg)	8.10
SO^2−^ (mg/kg)	74.50
PH	7.25
soluble salt (%)	1.61

**Table 2:** Surface macromorphology of galvanized steel.

Current Intensity	Original Sample	0 mA	0.5 mA	50 mA	300 mA
AC	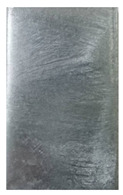	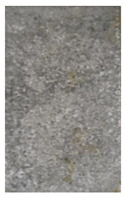	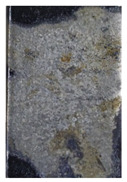	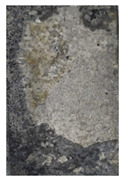	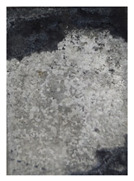
DC			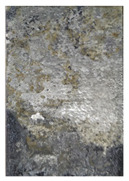	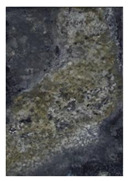	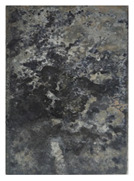

**Table 3:** Corrosion rate of galvanized steel.

Material	Number	Current mA	Weightlessness (W) g	Exposed Area (S) mm^2^	Weight Loss Rate (V) g/(mm^2^ × d)	Standard Deviation
Galvanized steel	1	0	0.0479	3573.542	0.9577	0.3556
	2	0.5, AC	0.2424	3595.879	4.8150	0.5328
	3	50, AC	0.2156	3602.056	4.2753	0.5512
	4	300, AC	0.3031	3687.286	5.8715	0.5104
	5	0.5, DC	0.27	3549.824	5.4329	0.5619
	6	50, DC	0.4347	3639.555	8.5313	0.6384
	7	300, DC	1.2357	3666.605	24.0725	0.6952
The current time (T): 14 days						

## Data Availability

Not applicable.
